# Using a Health Literacy Analytic Framework to Explore Zika Virus and Reproductive Health

**DOI:** 10.3928/24748307-20180226-01

**Published:** 2018-04-12

**Authors:** Erika L. Thompson, Cheryl A. Vamos, Langdon G. Liggett, Stacey B. Griner, Ellen M. Daley

## Abstract

**Background:**

The emergence of Zika virus as sexually transmissible and associated with birth defects may affect reproductive planning and contraception use for people in Florida.

**Objective:**

This exploratory study employed a health literacy analytic framework to explore knowledge, attitudes, beliefs, and behaviors related to reproductive health in the context of Zika among reproductive-age women and men in Florida.

**Methods:**

Reproductive-age people in Florida (*N* = 40) were interviewed between September and December 2016 about their knowledge, attitudes, beliefs, and behaviors regarding Zika and reproductive health. Thematic analysis using a health literacy framework was employed.

**Key Results:**

Participants reported they would use reputable online sources to access Zika information. Whereas participants generally understood Zika outcomes, transmission, and symptoms, they reported hearing more prevention messages on mosquito transmission compared to sexual transmission. Overall, participants reported Zika was not concerning given their appraisal of personal circumstances. Participants were confident they could prevent Zika via sexual transmission despite not following the recommended guidelines. Participants discussed how their understanding of Zika changed their behaviors related to mosquito control but not through sexual transmission.

**Conclusions:**

This study illustrated a disconnect between reproductive-age people's understanding of Zika-related prevention information and their reproductive decision-making behavior. Strategies to promote appraisal of risk for sexual transmission of Zika, infection, and unintended pregnancy are needed. **[*HLRP: Health Literacy Research and Practice.* 2018;2(2):e78–e87.]**

**Plain Language Summary:**

Men and women of reproductive age in Florida may be at risk for Zika virus and related negative health outcomes. This study assessed how Florida men and women find, understand, and evaluate Zika-related health information, and how that applies to their prevention behaviors. This study used health literacy as an analytic framework for an emerging health issue.

Zika virus spread to the United States, with most locally acquired cases (97%) occurring in Florida (Centers for Disease Control and Prevention, 2017b). Zika infection is primarily spread via mosquito transmission, but is also spread by sexual and maternal-fetal transmission. Although most cases of Zika infection are mild or asymptomatic, the virus is associated with severe secondary outcomes consisting of neurological disorders (Centers for Disease Control and Prevention, 2016). Of most concern is Zika's connection to a spectrum of congenital brain anomalies, including microcephaly (Honein et al. 2017). Among pregnant women in 2016 with confirmed Zika infections in the U.S., 1 in 10 had a fetus or baby with a birth defect (Reynolds et al., 2017). Although Zika was discovered in 1947, little was known about the virus until this recent outbreak, which began in Brazil in 2015, and in 2016 spread throughout the Americas (World Health Organization, 2017).

Scientific knowledge regarding transmission has evolved throughout the course of this emerging epidemic. Initially, Zika was thought to be transmitted only by mosquitos, but new evidence expanded knowledge about the modes of transmission. In January 2016, the Centers for Disease Control and Prevention (CDC) announced that the virus can be transmitted in-utero (Petersen, Staples, et al., 2016). Then, in February 2016, the CDC confirmed Zika can be transmitted sexually from males (Oster et al., 2016), and 5 months later, in July of 2016, the CDC announced Zika can be sexually transmitted from females to males (Davidson, Slavinski, Komoto, Rakeman, & Weiss, 2016). In response to these changes in knowledge, the CDC has issued evolving guidelines on preventing Zika via sexual transmission based on a person's (1) pregnancy intention, (2) gender, and (3) geographic location. Guidelines are intended to prevent unintended pregnancy and the maternal-fetal transmission of Zika, and they include recommendations on the use of effective contraceptive methods and condoms, pregnancy intention, and travel (Petersen, Meaney-Delman, et al., 2016).

As evolving knowledge of the Zika virus epidemic results in new preventive guidance, there is a need for accurate and timely information that is accessible and understandable by the public to help people make informed decisions, particularly in regard to reproductive health. One approach to addressing these prevention concerns for reproductiveage people is considering health literacy, which is a person's ability to make informed health decisions by accessing, understanding, appraising, and applying health information (Sørensen et al., 2012). In this context, health literacy is applied using a public health approach by recognizing the acquisition of health information, not just health knowledge (Pleasant & Kuruvilla, 2008). Health literacy is a key social determinant of health and is associated with a range of reproductive health issues (Kilfoyle, Vitko, O'Conor, & Bailey 2016) and, therefore, can be applied to this specific and complex reproductive health topic.

The CDC guidelines play an important role in containing the spread of Zika and preventing associated birth defects, but they are only as effective as they are successfully understood and applied by reproductive-age people in high-risk areas such as Florida. Specifically, in the United States, 22% of all Zika cases have occurred in Florida, and 97% of all locally acquired cases of Zika occurred in Florida (Centers for Disease Control and Prevention, 2017b). Locally acquired cases are infections deemed to be transmitted locally by mosquitos, and they indicate increased risk of infection for residents and visitors to affected areas. An area of active transmission is declared when two locally acquired cases occur in an area within 45 days of one another (Centers for Disease Control and Prevention, 2017a). Although Miami-Dade County and Palm Beach County were the only counties in Florida to have active transmission of Zika in 2016, travel-related cases were documented in most counties across the state (Florida Department of Health, 2017), which may increase the risk for infection via sexual and mosquito transmission for residents and visitors (Armstrong et al., 2016; Centers for Disease Control and Prevention, 2017c).

An additional concern related to Zika is the risk of unintended pregnancy and potential for birth defects. In Florida, 59% of pregnancies are unintended, which is higher than the national average (Kost, 2015). This can be attributed to the low rate of effective contraceptive use. Specifically, more than one-quarter (27.5%) of women age 18 to 44 years at risk for unintended pregnancy in Florida were not using any form of contraception, and only 6.8% used a highly effective, reversible form of contraception (Boulet et al., 2016). The risk of unintended pregnancy, combined with the risk for Zika infection, creates an exceptionally volatile environment concerning Zika-related birth defects. Counties throughout the southeastern region of the U.S., where sexually transmitted infection rates are high and mosquitos are prevalent, are especially high-risk areas for Zika transmission, indicating the need for targeted prevention (Shacham, Nelson, Hoft, Schootman, & Garza, 2017). Complicating this prevention issue, the periconceptional period is the time for highest risk of birth defects—a time when a woman may not know she is pregnant (Honein et al., 2017). Moreover, men can play an active role in Zika prevention by limiting sexual transmission and preventing unintended pregnancies through birth control decision-making and negotiation with partners (Osamor & Grady, 2016).

Given the new evidence that Zika can be transmitted through sexual activity and the resulting risk of birth defects, prevention guidelines for Zika are critical for reproductiveage people in Florida. Assessing the Zika-related health literacy among this target population is essential to inform effective health communication strategies that aim to prevent Zika infection. Thus, the objective of this exploratory study was to employ a health literacy analytic framework to explore knowledge, attitudes, beliefs, and behaviors related to reproductive health in the context of Zika among reproductive-age women and men in Florida.

## Methods

The target population for this study was men and women of reproductive-age (age 18–44 years) residing in the state of Florida. Participants were recruited via online social media postings (Facebook and Twitter) and on campus flyers at a Florida university between September and December 2016. It should be noted that the study began with exclusive social media recruitment for the first 30 participants and then expanded to social media and campus flyers for the remaining 10 participants. Interested people completed an eligibility questionnaire. Inclusion criteria were (1) age 18 to 44 years, (2) residing in the state of Florida, and (3) being sexually active. Persons were excluded if they were currently pregnant or had a partner that was currently pregnant. The convenience sample comprised 40 men and women. This study was approved by the University of South Florida Institutional Review Board.

This was a concurrent mixed-methods study that used (1) a quantitative survey to ascertain participant demographics, individual behavior, and Zika knowledge; and (2) a qualitative semistructured interview to elicit participants' attitudes and beliefs regarding Zika and reproductive health. Participants were contacted via email to schedule a telephone interview and provided with an online survey link to complete a preinterview questionnaire. The preinterview questionnaire included demographic questions such as sex (male, female), age, county of residence, Hispanic ethnicity (yes, no), race (Black, White, Multi-/Biracial, Other), sexual orientation (heterosexual, lesbian, gay, bisexual), and relationship status (married/civil union, committed relationship, single). County of residence was classified as having active Zika transmission if two locally acquired cases occurred in an area within 45 days of one another (Centers for Disease Control and Prevention, 2017a). Additionally, participants reported on sexual activity in the last 12 months (vaginal, oral, anal sex), condom use at last sexual encounter, and contraceptive use at last sexual encounter. The type of contraceptive used was classified as highly effective (female sterilization, tubal ligation, vasectomy, intrauterine devices, and contraceptive implants), moderately effective (hormone injections, contraceptive pills, transdermal contraceptive patches, and vaginal rings), and less effective (diaphragm, male condoms, female condoms, cervical cap, contraceptive sponge, withdrawal, spermicide, fertility-based awareness methods, emergency contraception, and other) (Boulet et al., 2016). Pregnancy intention in the next year was assessed using a statement and a question: “Getting pregnant right now would be good for me,” which used a 5-point Likert scale from *strongly agree* to *strongly disagree* (cognitive), and “How much do you want to get pregnant in the next year?”, which used a 10-point Likert scale from *don't want to get pregnant at all* to *very much want to get pregnant* (affective). Participants were categorized as either intending pregnancy, intending to avoid pregnancy, or ambivalent/inconsistent.

Participants responded to questions related to Zika virus, including 10 knowledge items, using a true/false format. This was constructed using CDC guidance and their website for information regarding prevention, transmission, and outcomes associated with Zika virus to ensure content validity. Participants who had missing responses to these knowledge questions were coded as incorrect/unsure as a response. Finally, participants were asked if they would change their birth control and sexual behavior, respectively, due to Zika in their area. Participants responded yes or no. All quantitative responses were aggregated from the online survey. Frequencies and proportions were calculated using Microsoft Excel. The telephone interviews used a semistructured interview guide and lasted approximately 30 minutes. The semistructured interview guide was designed to acquire participants' knowledge, attitudes, beliefs, and behaviors regarding Zika virus and reproductive health. First, participants gave their consent and then were asked to describe what they knew about Zika virus, including probes on transmission, outcomes, areas of transmission, and prevention messages. Then the interviewer read the CDC statement on guidance for prevention of sexual transmission of Zika for couples not intending to be pregnant (Brooks et al., 2016). Participants were asked about confidence preventing Zika, trusted information sources for Zika virus, and questions for a health care provider about Zika virus and reproductive health. During these responses, participants disclosed their current prevention behaviors for Zika virus. All interviews were audio recorded and transcribed verbatim.

Interviews were analyzed using thematic analyses. Interviews were coded using *a priori* codes based on the semi-structured interview guide. The codebook was developed through an iterative process with the research team (Bernard & Ryan, 2010). Two members of the research team conducted open coding on 10% of the transcript sample and reached an inter-rater reliability level indicating strong agreement (kappa = 84%). Once reliability was established, one member of the research team conducted the open coding of the remaining transcripts.

Upon completion of coding, themes and relationships among codes were identified through axial coding (Bernard & Ryan, 2010). These emergent codes were relevant to the constructs of health literacy (Sørensen et al., 2012). Therefore, we used the four overarching constructs of health literacy as defined by the European Health Literacy Framework (access, understand, appraise, and apply) as the analytic framework for this study to identify the meta-themes **(Table 1**). These four overarching themes guided the analysis for this study, and the results of this study are presented by each health literacy meta-theme. Summaries of the meta-themes were written and representative quotes chosen; both were agreed upon by the research team. This analysis revealed data redundancy was reached for these meta-themes using the health literacy framework.

## Results

Of the 40 participants, the majority were women (*n* = 34), White (*n* = 34), non-Hispanic (*n* = 36), heterosexual (*n* = 31), and married or in a civil union (*n* = 19) (**Table 2**). Additionally, all but one of the participants resided in a Florida county with confirmed cases of Zika, and two participants resided in a Florida county with active Zika transmission. Regarding sexual behavior, 27 participants did not use a condom at last vaginal intercourse, and only 12 used a highly effective form of contraception (i.e., sterilization, intrauterine device, implant). Most participants wanted to avoid pregnancy in the next year (80%).

### Access

Participants were asked where they would go to find information about Zika. Most participants mentioned the CDC (*n* = 36), and stated that they considered online sources that are funded by the government to be trustworthy and reputable. Most participants favored online searches, as opposed to in-person discussions or more traditional news sources.

Honestly I would probably go to just, like, Google ‘Zika’ and then try to find somewhere with dot-GOV domain and read some information from a government website.

(Female, age 20)

I would want to go to straight facts. I would go to the National Institutes of Health or the CDC or researchers or departments that really know what they're doing with this. I wouldn't go to yahoo.com where you ask a question and they reply. If I was super concerned, I would also consult with my doctor as well.

(Female, age 19)

The CDC is largely a trusted source for me. And then I often will consult– for general information… just kind of a consensus of reviewing like web literature, like WebMD, that kind of thing. But if it's something I'm really curious about or skeptical about, I'll [consult] the literature directly.

(Male, age 33)

### Understand

In the survey, most participants reported knowing *somewhat* (*n* = 18) or *a little* (*n* = 17) about Zika. In assessing Zika virus knowledge, the most frequently missed question was whether condoms can be used to prevent Zika (*n* = 17) **(Table 3**). Most participants knew that Zika is sexually transmitted (*n* = 34) and correctly marked as false the statement that Zika always causes symptoms when someone is infected (*n* = 30).

During the interview, when asked what they knew about Zika, most participants knew that it was a virus transmitted by mosquitos (*n* = 36), sexually transmitted (*n* = 31), and linked to birth defects (*n* = 32). Most who stated knowing about both forms of transmission were certain about mosquitos, but less certain about stating it was sexually transmitted.

Well it is a virus that is transmitted by mosquitos or perhaps even sexually. If a woman who is pregnant gets the virus it can affect the baby in a way that is devastating … it can cause a problem with the skull and the head where it's a smaller size, obviously have mental problems after that, that's about what I know.

(Male, age 37)

So the prevention messages I've seen have been about trying to get rid of mosquitos, so like getting rid of standing water and wearing mosquito repellent.

(Female, age 35)

I know that it's transmitted by mosquitos and that it can also be sexually transmitted and that it's a generally pretty mild illness, but that it can cause birth defects if women are pregnant when they contract it, microcephaly, mainly.

(Female, age 26)

Actually, so I wasn't aware up until recently, I have a friend who informed me that it can also be sexually transmitted. I had no idea up until 2, 3 weeks ago, we were having a discussion about it and they said that men that it could be in their sperm and it could be transmitted that way.

(Female, age 31)

Only a few participants reported misinformation about Zika, including airborne transmission, only transmitted via blood, and no link of Zika to microcephaly.

To identify areas of knowledge gaps, participants were asked what questions they would have for a health care provider regarding Zika. Questions often centered on assessing risk for infection and transmission (*n* = 11), ways of prevention and testing for Zika (*n* = 22), and the health outcomes associated with people and fetuses exposed to Zika (*n* = 23).

I guess maybe just knowing more about specifics instead of it being so broad about–is it every type of sexual act? What is the percentage of you actually getting it by doing that? Again, is [sic] there certain types of prevention that actually could be more effective than others?

(Female, age 31)

You know since I'm not planning to get pregnant I think it [questions/concerns] would just be, should I be concerned about this? Should my husband and I be using condoms just from the chance that we do get it from a mosquito? Or is it something that a healthy person is going to get and it'll just go away and it won't be a big deal? Should we use condoms to prevent the virus given that we aren't going to have a baby?

(Female, age 40)

### Appraise

Appraisal in the health literacy framework reflects a person's ability to evaluate the health information obtained. This can include judging how this information applies to personal circumstances or risk. In the context of Zika virus and reproductive decision-making, the appraisal process included factors such as confidence preventing Zika and risk perceptions for Zika.

Participants were asked how confident they were in their ability to prevent Zika infection. Most participants were confident in their capacity to prevent infection by sexual transmission (*n* = 31), and perceived their risk for sexual transmission to be low, mainly due to monogamy. Most participants were not confident in their capacity to prevent infection by mosquito transmission (*n* = 20), stating that mosquito bites were unavoidable in Florida, in spite of any preventive measures.

Because I don't perceive the sexually transmitted aspect of disease to be the risk factor for me. I see more of the vector borne aspect of it as problematic, and I feel like I don't really have a lot of control over what mosquito has it or doesn't.

(Male, age 33)

Well, I think I can minimize it, but I'm more concerned about transmitting it–well, I could certainly get it from my husband, but it's–I feel more susceptible to getting it from a mosquito than I do through sexually transmitted–I guess that's my point.

(Female, age 43)

Well, I don't think you can ever entirely prevent a disease transfer by a tiny bug in Florida. They are everywhere.

(Female, age 32)

I guess I feel pretty confident about it because–again, I don't know fully everything. I don't know if putting on a repellant would be–I'm really not sure about that. Again, as far as the sexual part, I'm not really active right now. I don't have a lot of partners so as far as that goes, I feel very confident in that.

(Female, age 31)

If we're limiting [preventing Zika] to sexually transmitted Zika infection, then I would say that I'm very confident that I can. If we're…just saying in general, I have very low confidence, because I just think my personal mosquito prevention methods are inadequate.

(Male, age 33)

Most stated that Zika did not apply to their personal health, supplementing with justifications such as they are currently not pregnant or not intending to be pregnant, already using what they perceive as effective contraceptive methods, in a monogamous relationship, and not at high risk. Few people said that Zika affects their health (*n* = 4), citing the sexual transmission, the unknowns, and wanting to get pregnant in the future.

A couple that's monogamous–I don't feel [they] need to take extra protection against Zika.

(Female, age 37)

If you're using condoms properly, you're not at super high risk for contracting Zika through sex, and so the additional birth control is not something that someone should do because of Zika.

(Male, age 33)

### Apply

Participants described their changes in behavior due to Zika. This was limited to prevention of mosquito bites through bug spray (*n* = 8), removing standing water (*n* = 2), and changing the time of day they are outside (*n* = 5). A majority of participants stated they did not change any prevention behaviors in response to Zika (*n* = 35). Most participants stated they would not change their birth control methods (*n* = 25) or sexual behaviors (*n* = 29) because of Zika in their area.

Well just doing the normal stuff that we are doing regularly as far as you know spraying ourselves with mosquito repellent and not leaving any water standing around the house and you know we are committed to each other so you know being monogamous in our relationship is what we're doing.

(Female, age 42)

I'm already using a contraceptive method. I'm already avoiding getting pregnant. I'm already in a long-term relationship, so most of my habits would stay constant.

(Female, age 20)

It's one of those things that I'm not really concerned if I get infected, I guess. Again, I think that I would be more concerned if I was trying to conceive a child, but I'm not really concerned since I'm not trying to conceive a child.

(Male, age 35)

## Discussion

This formative study assessed health literacy related to reproductive health and Zika virus in Florida. Although participants reported adequate abilities to access and understand Zika-related prevention information, there appeared to be difficulty in evaluating how to use this information. Moreover, most behavior change in response to Zika virus was related to mosquito control rather than sexual transmission advisories.

Participants in this study stated the most trusted information source for Zika information was the CDC website, which currently offers a wide range of public and health care provider education materials (Centers for Disease Control and Prevention, 2016). Trust in government organizations has not always been an asset during health outbreaks. For example, a study among college students found low use of government websites during the Ebola outbreak compared to the news media (Koralek, Runnerstrom, Brown, Uchegbu, & Basta, 2016). The confidence in the CDC website during the Zika virus may be the result of the CDC's rapid information dissemination during the outbreak (Iskander, Rose, & Ghiya, 2017). A content analysis of a Zika Twitter chat with the CDC illustrated the public's engagement with the CDC as a source for Zika information, specifically as it relates to consequences of Zika for pregnant women and babies (Glowacki, Lazard, Wilcox, Mackert, & Bernhardt, 2016). Additionally, most participants in this study stated they preferred using online resources to access information regarding the virus and prevention. This proclivity for online governmental websites may be attributable to the sample characteristics and recruitment of some participants through social media. An assessment of obstetric practice websites found that 35% of websites sampled included information on Zika virus in August 2016 and primarily focused on insect prevention and travel recommendations (Lehnert et al., 2017). Public health efforts focused on reaching people of reproductive age in Florida for Zika prevention should use the information sources identified in this current study presented here, such as government websites, to reach similar audiences, and they should include a range of prevention messages beyond mosquito transmission. Previous research among young adults indicates online sources as an acceptable information channel for health topics (Briones, 2015), including sexual health (Buhi, Daley, Fuhrmann, & Smith, 2009). These young adults are a large proportion of the reproductive-age population considered for Zika prevention behaviors. This will require harnessing e-health literacy skills to promote online health information-seeking behaviors (Norman & Skinner, 2006).

Although the focus of this study was on the sexual transmission of Zika virus, mosquito risk reduction is also essential to prevention. Yet, because these participants resided in Florida, there appeared to be a sense of fatalism regarding mosquito bites. Although this study identified the county of residence for participants, this does not consider daily, recreational, or work-related travel. Previous research found that persons were more likely to avoid travel to Florida if they placed a high value on their health (Widmar, Dominick, Ruple, & Tyner, 2017). Coherent public health messages need to continue to emphasize the importance of personal protective behaviors for prevention of mosquito bites, as well as to make the public aware of travel advisories to areas where Zika is prevalent.

Overall, participants in this study had adequate knowledge on the basics of Zika virus and prevention. In comparison, a study with a university sample found students who had higher knowledge of Zika and knew where to get information were more likely to be accepting of a hypothetical Zika vaccine for prevention (Painter, Plaster, Tjersland, & Jacobsen, 2017). In this study, although participants understood ways to prevent the sexual and mosquito transmission of Zika separately, there was difficulty connecting these two forms of transmission. Specifically, participants reported that monogamy was a protective factor for reducing the risk of Zika transmission; yet, in the case of Zika, monogamy does not reduce the risk of transmission as partners can be infected by a mosquito. This virus is unlike other sexually transmitted diseases in which monogamy is a risk-reduction behavior (Aral & Leichliter, 2010). The emergence of Zika may require a change in risk and prevention strategies for a sexually transmitted virus that can also be spread by mosquitos. Moreover, given this sample's low risk perceptions for Zika, desire to avoid pregnancy, and lack of barrier method use, an emphasis on using highly effective reversible contraceptive methods to prevent unintended pregnancy is needed.

Zika risk perceptions among this sample were low. This perception of low Zika risk could be why a small proportion of participants said they would change their sexual behaviors or birth control methods in response to Zika presence in their physical area. Similarly, in a June 2016 poll of U.S. adults, among those who knew Zika was transmitted sexually, 12% changed their sexual behavior due to Zika and 7% had or planned to change their birth control because of Zika (The National Campaign to Prevent Teen and Unplanned Pregnancy, 2016). These low-risk perceptions of Zika are contrasted with the potential actual risk for Zika infection. Most participants reported not using a barrier method at last vaginal sex and using less or moderately effective forms of contraception. Moreover, many participants resided in counties of Florida with active or travel-associated transmission of Zika. Recognizing risk perceptions as a barrier to behavior change can inform the health communication messages needed to promote Zika prevention. Thus, improved health literacy efforts are needed to show this connection between reproductive health behaviors and Zika and to adapt this information to personal circumstances. Such efforts should provide reproductive-age men and women with easy-to-access information that is presented using health literacy principles (e.g., lay language) (Brega et al., 2015). This information could also be incorporated into patient-centered decision-aid tools, which help people evaluate their risks according to their personal circumstances (e.g., sexual behaviors, pregnancy intention) and provide the skills necessary to engage in the appropriate preventive behaviors. This study used the European Health Literacy Framework to examine individual-level health literacy skills of accessing, understanding, appraising, and applying health information (Sørensen et al., 2012). In addition to exploring these competencies for health literacy, an exploration of the systemic issues of the situational and environmental determinants to Zika prevention and reproductive health is needed. These situational and environmental determinants can include the availability and access to contraception, condoms, or mosquito repellant, and other sociocultural determinants of pregnancy and family planning (Rasanathan, MacCarthy, Diniz, Torreele, & Gruskin, 2017).

These findings should be considered within the context of the limitations. First, this study used a small convenience sample for this formative investigation. The types of participants and smaller proportion of males in the sample may not provide the full range of perspectives regarding Zika prevention and Zika-related health literacy. Guest, Bunce, and Johnson (2006) indicate that data saturation can be reached at approximately 12 interviews, and meta-themes apparent at 6 interviews. In this context, a sufficient sample size was reached; however, data redundancy was achieved rather than saturation (Guest, et al. 2006). This may be attributed to the heterogeneity of the sample in terms of contraceptive practices, access to health services, socioeconomic status, and other contextual factors. In addition, the study used two strategies for recruitment—social media (used exclusively for the first 30 participants) and university campus flyers. As a result, the educational attainment and field of study of our sample may be different than the general population; however, this information was not collected in the study to assess this as a factor. Future research should conduct this study among a larger, more generalizable sample of reproductive-age persons in Florida while considering stratifications by sex and other contextual factors in the sample. Additional research may reveal other information sources and channels for public health messages beyond online resources. Moreover, other recruitment strategies should be employed beyond online recruitment, as this may have attributed to the participants' favoring online government sources for information.

Additionally, the theoretical framework for this analysis (ie, health literacy) was not used *a priori* for data collection. However, the emergence of these themes was clear from the results and provides an example of how health literacy can be critical for an emerging health issue. Future research could also expand to other aspects of information-seeking, such as where people could go for information, in addition to where they do go for information. Finally, there is the possibility for social desirability bias in the report of prevention and sexual health behaviors. To reduce this type of bias, the interviews were conducted via telephone, and more sensitive questions were asked via an online survey. The quantitative survey online permitted participants to skip questions, which resulted in missing data for some of the questions.

This is one of the few studies to examine reproductive health in the context of Zika among a reproductive-age, non-pregnant population. It is clear that despite media and health officials' dissemination of health messages, health literacy approaches are required to promote behavior change to prevent Zika infection. Given Zika virus' potential to severely affect reproductive health, this is an area of investigation that is needed as Zika continues to occur in Florida and the U.S.

With Zika virus' rapid emergence as a public health issue, this case study highlights the need to integrate health literacy tenets from the onset of a health crisis. Integrating health communication and health literacy principles in the dissemination of prevention information regarding a new phenomenon is needed to promote understanding and use of that information by the public (Parson, Allen, Alvarado-Little, & Rudd, 2017). The extent to which critical information regarding emerging health threats is accurately and effectively communicated to the public may influence the impact and scope of the problem, particularly in the initial stages. Findings from this study can highlight lessons learned from the Zika virus crisis, and can be applied to future reproductive health threats and concerns.

## Figures and Tables

**Table 1:** Health Literacy Definitions for Analytic Framework

**Construct**	**Definition^a^**	**Application to Zika**
Access	Ability to find information	Information-seeking related to Zika prevention
Understand	Ability to comprehend information	Understanding of Zika and Zika prevention Participants' questions about Zika and reproductive health
Appraise	Ability to evaluate health information	Confidence preventing Zika sexually and by mosquitos Zika's influence on reproductive health plans and contra-ceptive methods
Apply	Ability to communicate or use health information	Participants' prevention behaviors for Zika virus

a Based on the definition of health literacy by Sørensen et al. (2012).

**Table 2:** Demographic Characteristics (*N* = 40)

**Variable**	***n* (%)**

Gender	
Female	34 (85)
Male	6 (15)

Race	
White	34 (85)
Black	3 (7.5)
Biracial/multiracial	2 (5)
Other	1 (2.5)

Hispanic ethnicity	4 (10)

Sexual orientation	
Heterosexual	31 (77.5)
Bisexual	7 (17.5)
Lesbian/gay	2 (5)

Relationship status	
Married/civil union	19 (47.5)
Committed relationship	17 (42.5)
Single	4 (10)

County of residence (number of travel-related cases in 2016)	
Hillsborough (44)	18 (45)
Pinellas (25)	6 (15)
Alachua (11)	2 (5)
Miami-Dade (332)	2 (5)
Orange (158)	2 (5)
Pasco (9)	2 (5)
Polk (31)	2 (5)
Seminole (27)	2 (5)
Collier (27)	1 (2.5)
Osceola (38)	1 (2.5)
Putman (0)	1 (2.5)
Sarasota (5)	1 (2.5)

Active transmission in county (2016)	
No	38 (95)
Yes	2 (5)

Condom use at last intercourse	
No	27 (67.5)
Yes	11 (27.5)
Response missing	2 (5)

Effectiveness of contraceptive method	
Less	15 (37.5)
Moderate	12 (30)
High	12 (30)
Response missing	1 (2.5)

Pregnancy intention	
Avoid pregnancy	32 (80)
Desires pregnancy	4 (10)
Inconsistent intention	2 (5)
Response missing	2 (5)

**Table 3:** Knowledge Questions Related to Zika (*N* = 40)

	**True/False Items**	**Correct, *n* (%)**	**Incorrect/Unsure, *n* (%)**
	Zika is sexually transmitted	34 (85)	6 (15)
	Zika is transmitted by a mosquito	36 (90)	4 (10)
	Zika can cause birth defects	36 (90)	4 (10)
	Condoms can be used to prevent Zika	23 (57.5)	17 (42.5)
	Zika always causes symptoms when someone is infected^a^	30 (75)	10 (25)
	Men can transmit Zika to their partners	32 (80)	8 (20)
	Women can transmit Zika to their partners	28 (70)	12 (30)
	There is a vaccine available that can prevent Zika^a^	25 (62.5)	15 (37.5)
	There is a test to screen for Zika infection	26 (65)	14 (35)
	Wearing mosquito repellent can prevent Zika	30 (75)	10 (25)

a False is the correct answer.
